# Epidemiology and specific features of shoulder injuries in patients affected by epileptic seizures

**DOI:** 10.1007/s00402-022-04420-6

**Published:** 2022-03-28

**Authors:** Davide Cucchi, Tobias Baumgartner, Sebastian Gottfried Walter, Alessandra Menon, Robert Ossendorff, Rainer Surges, Christof Burger, Dieter Christian Wirtz, Max Julian Friedrich

**Affiliations:** 1 Department of Orthopaedics and Trauma Surgery, University Hospital Bonn, Venusberg-Campus 1, 53127 Bonn, Germany; 2 Department of Epileptology, University Hospital Bonn, Venusberg-Campus 1, 53127 Bonn, Germany; 3 Department for Orthopedic Surgery and Traumatology, University Hospital Cologne, Kerpener Str. 62, 50937 Köln, Germany; 4 ASST Gaetano Pini-CTO, Piazza Cardinal Ferrari 1, 20122 Milan, Italy; 5 Laboratory of Applied Biomechanics, Department of Biomedical Sciences for Health, Università degli Studi di Milano, Via Mangiagalli 31, 20133 Milan, Italy; 6 Scuola di Specializzazione in Statistica Sanitaria e Biometria, Dipartimento di Scienze Cliniche e di Comunità, Università degli Studi di Milano, Via Mangiagalli 31, 20133 Milan, Italy

**Keywords:** Seizure, Epilepsy, Dislocation, Instability, Posterior, Bilateral

## Abstract

**Purpose:**

Epileptic seizures can cause multiple shoulder injuries, the most common of which are dislocations, recurrent instability, fractures, and isolated lesions of the rotator cuff. Currently, only limited literature exists which describes the frequency and types of lesions in cohorts of epileptic patients and the corresponding treatment outcome. This study aims to document the occurrence of shoulder lesions in patients affected by seizures and to provide detailed information on trauma dynamics, specific lesion characteristics and treatment complications.

**Methods:**

All patients referring to a tertiary epilepsy center were screened for shoulder injuries and the clinical records of those sustaining them during a seizure were reviewed. Demographic information, lesions’ characteristics and trauma dynamics were analysed, as wells as—when carried out—the type of surgical intervention and any postoperative complications.

**Results:**

The average age at the time of injury of 106 included patients was 39.7 ± 17.5 years and a male predominance was recorded (65%). Bilateral injuries occurred in 29 patients, simultaneously in 17 cases. A younger age, bilateral shoulder injuries and shoulder dislocations were significantly associated with the occurrence of a shoulder injury solely by muscular activation (*p* = 0.0054, *p* = 0.011, *p* < 0.0001). The complication rate in 57 surgically treated patients with follow-up data was 38.7%, with recurring instability being the most frequently reported complication (62.5%).

**Conclusions:**

Uncontrolled muscle activation during a seizure is a distinctive but not exclusive dynamic of injury in epileptic patients, accounting for more than the half of all shoulder lesions, especially in the younger. This can lead both to anterior and posterior dislocations or fracture-dislocations and is frequently cause of bilateral lesions and of instability recurrence after surgery. The high complication rates after surgical treatment in this selected subgroup of patients require that appropriate preventative measures are taken to increase the probability of treatment success.

**Level of evidence:**

Cohort study, level III.

**Supplementary Information:**

The online version contains supplementary material available at 10.1007/s00402-022-04420-6.

## Introduction

Musculoskeletal injuries are a well-known but rather neglected complication of epileptic seizures and particularly tonic–clonic seizures; they significantly reduce the quality of life for the affected patients. Despite growing scientific interest, the number of studies addressing these injuries and their risk factors is limited. Among the vast spectrum of different musculoskeletal injuries, shoulder injuries account for a major percentage; these include fractures of the humerus, scapula or clavicle, acute or recurrent shoulder dislocations, rotator cuff lesions, or a combination of these injuries [1, 2]. Detailed descriptions of specific shoulder lesion characteristics are difficult to obtain from large cohorts or cross-sectional studies which focus primarily on other epidemiological aspects; these lesions are included in broader categories such as, “upper extremity injuries” or “other fractures”. In contrast, the few studies dedicated to the orthopedic treatment of shoulder injuries in patients affected by seizures are abundant in terms of details, but limited sample size and lacking in information concerning the neurological history [3–8]. The aim of this study was to analyse the epidemiology of a large cohort of patients suffering from seizure-related shoulder injuries and to provide detailed information on trauma dynamics and specific lesion characteristics.

## Methods

The goal of this study was to describe the epidemiology and the specific features of shoulder injuries occurring during seizures in a selected subgroup of patients with epilepsy.

A screening for upper limb injuries was performed on all patients referring to a tertiary epilepsy center from January 2015 to June 2020 with an anamnestic questionnaire completed at the time of stationary admittance or during outpatient consultations, under the supervision of a clinician. For patients ruled in during this screening phase, relevant clinical and radiological records were collected and reviewed to investigate dynamics and characteristics of shoulder injuries.

Adult patients suffering from a bony or soft tissue shoulder injury sustained during a seizure were considered for inclusion. Patients with injuries sustained in paediatric age, caused by other aetiologies than a seizure or affecting other regions of the body were excluded.

Data collected during clinical care were integrated by telephonic interviews to gather full information on the injury in terms of trauma dynamics, bone or soft tissue involvement, affected side, as well as the type and direction of instability for glenohumeral dislocations. If surgical treatment was required, the type of intervention and possible subsequent complications were recorded.

Demographic data, as well as data regarding diagnosis and treatment of epilepsy and information on shoulder injuries, were entered into a spreadsheet for analysis. Statistical analysis was performed using GraphPad Prism v 6.0 software (GraphPad Software Inc.). The Shapiro–Wilk normality test was used to evaluate the normal distribution of the sample. Continuous variables were expressed as the mean ± standard deviation (SD) or medians and first and third quartiles [Q1–Q3], as appropriate. The differences between the groups of patients for continuous variables were evaluated with unpaired Student’s *t* test or Mann–Whitney test according to the characteristics of the data distribution. Categorical variables are expressed in numbers of cases and frequencies; their differences were tested using with the chi-squared test or Fisher’s exact test. Variables significant at univariate analysis were inserted in a multivariable logistic regression model to correct for confounding and avoid multiple test correction and to estimate multivariate odds ratios (ORs) for evaluating the association between covariates and development of recurrent instability, of perioperative complications and of recurrent instability as a complication after surgical treatment. For all analyses, the significance level was set at *p* value lower than 0.05.

This audit of data collected during clinical care was approved by the local medical ethics committee (Ethikkommission an der Medizinischen Fakultät der Rheinischen Friedrich-Wilhelms-Universität Bonn, No. ID 245/19).

## Results

### Demographics, clinical history, and trauma dynamics

**Fig. 1:**
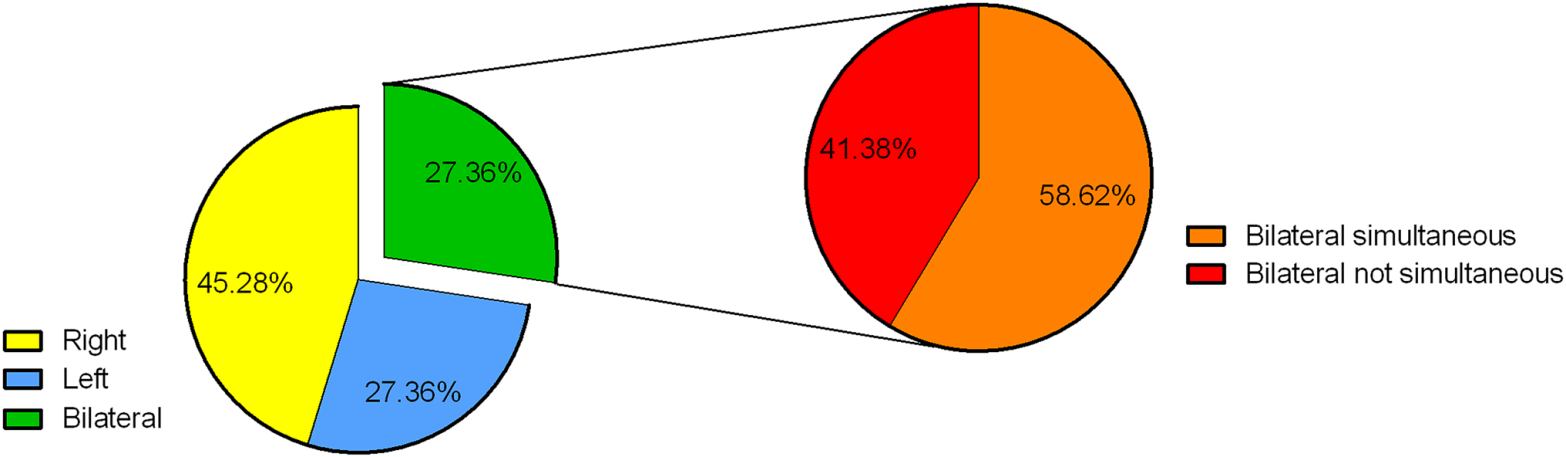
Pie charts illustrating the affected side and the distribution of bilateral lesions in the study cohort

Approximately 15,000 clinical consultations were evaluated in the screening phase and 106 patients with shoulder injuries met the inclusion criteria. Baseline neurological diseases included focal epilepsies (45%), unknown epilepsy types (32%), genetic generalized epilepsies (19%), and acute symptomatic seizures (4%). Average age at the time of injury was 39.7 ± 17.5 years and a male predominance was recorded (65%). No significant differences were encountered in the average age of the male and female patients. Bilateral injuries occurred in 29 patients (27.4%) and, of those, 17 patients had bilateral shoulder injuries which occurred simultaneously (Fig. 1). In 23.6% of the cases, shoulder injury occurred during the first seizure. At the moment of injury, 52.8% were not taking any antiepileptic drugs (AEDs), either because their epilepsy was not yet diagnosed, or because the medication had been discontinued due to low patient compliance, or a medical withdrawal from the medication was attempted.

A description of the trauma dynamics derived from direct observation by witnesses or during in-hospital monitoring was available for 76 patients (Table 1); in 47 cases (61.8%), the shoulder injury was caused solely from the muscular activation during the seizure without any external acting force or fall. In the remaining 29 cases (38.2%), the shoulder injury was caused by uncontrolled falls with direct impact on the shoulder joint. No patients sustained a shoulder lesion after a fall on the outstretched hand. The subgroup of patients experiencing shoulder injuries due to muscular activation was younger at the time of injury (*p* = 0.0054). Bilateral shoulder injuries and shoulder dislocations occurred much more frequently within the subgroup of patients experiencing shoulder injuries due to muscular activation (*p* = 0.011 and *p* < 0.0001, respectively), whereas fractures occurred much more frequently within the subgroup of patients experiencing shoulder injuries due to uncontrolled falls with impact on the shoulder (*p* = 0.0149).

**Table 1:** Trauma dynamics in the study population

Group	Overall	Muscular activation alone	Uncontrolled fall on the shoulder	*p* value
No. of patients	**76**	**47**	**29**	
Gender (F/M ratio)	0.33/0.67	0.34/0.66	0.31/0.69	1.0000 (n.s.)
Age at time of shoulder injury (years)	38.00 [27.00–55.00] 40.83 ± 18.39	35.00 [23.00–47.00] 36.34 ± 16.84	46.50 [32.00–60.75] 48.36 ± 18.70	0.0054 (**)
Shoulder injury during 1st seizure (Y/N ratio)	0.27/0.73	0.28/0.72	0.25/0.75	1.0000 (n.s.)
AED at shoulder injury time (Y/N ratio)	0.49/0.51	0.48/0.52	0.52/0.48	0.8108 (n.s.)
Side (L/R ratio)	0.35/0.65	0.37/0.63	0.32/68	0.7756 (n.s.)
Bilateral/unilateral lesions ratio	0.32/0.68	0.43/0.57	0.14/0.86	0.0110 (*)
Rotator cuff tears (Y/N ratio)	0.18/0.82	0.23/0.77	0.10/0.90	0.2254 (n.s.)
Shoulder instability (Y/N ratio)	0.71/0.29	0.89/0.11	0.41/0.59	< 0.0001 (****)
Single dislocation/recurrent shoulder instability (ratio)	0.52/0.48	0.45/0.55	0.25/0.75	0.1029 (n.s.)
Pure anterior dislocation/shoulder dislocation with posterior component (ratio)	0.59/0.41	0.57/0.43	0.67/0.33	0.7419 (n.s.)
Any fracture (Y/N ratio)	0.61/0.39	0.49/0.51	0.79/0.21	0.0149 (*)
Proximal humerus fractures (Y/N ratio)	0.54/0.46	0.47/0.53	0.66/0.34	0.1557 (n.s.)
Scapular fractures (Y/N ratio)	0.08/0.92	0.06/0.94	0.10/0.90	0.6688 (n.s.)
Clavicle fractures (Y/N ratio)	0.09/0.91	0.04/0.96	0.17/0.83	0.0984 (n.s.)
Shoulder fracture-dislocation (Y/N ratio)	0.36/0.64	0.40/0.60	0.28/0.72	0.3267 (n.s.)
No. of patients	**54**	**38**	**16**	
Complications after surgery (Y/N ratio)	0.39/0.61	0.45/0.54	0.75/0.25	0.2287 (n.s.)
Recurrent shoulder instability after surgery (Y/N ratio)	0.22/0.78	0.32/0.68	0/1	0.0110 (*)

Continuous variables were expressed as mean ± standard deviation (SD) or as median and interquartile range (first and third quartiles, Q1–Q3), as appropriate, while the dichotomous variables are expressed in frequencies

Bold values indicate* p* < 0.5

*AED* antiepileptic drug; *F/M* female/male; *L/R* left/right; *n.s.* not significant; *Y/N* yes/no

### Specific injuries subgroups and treatment results

**Fig. 2:**
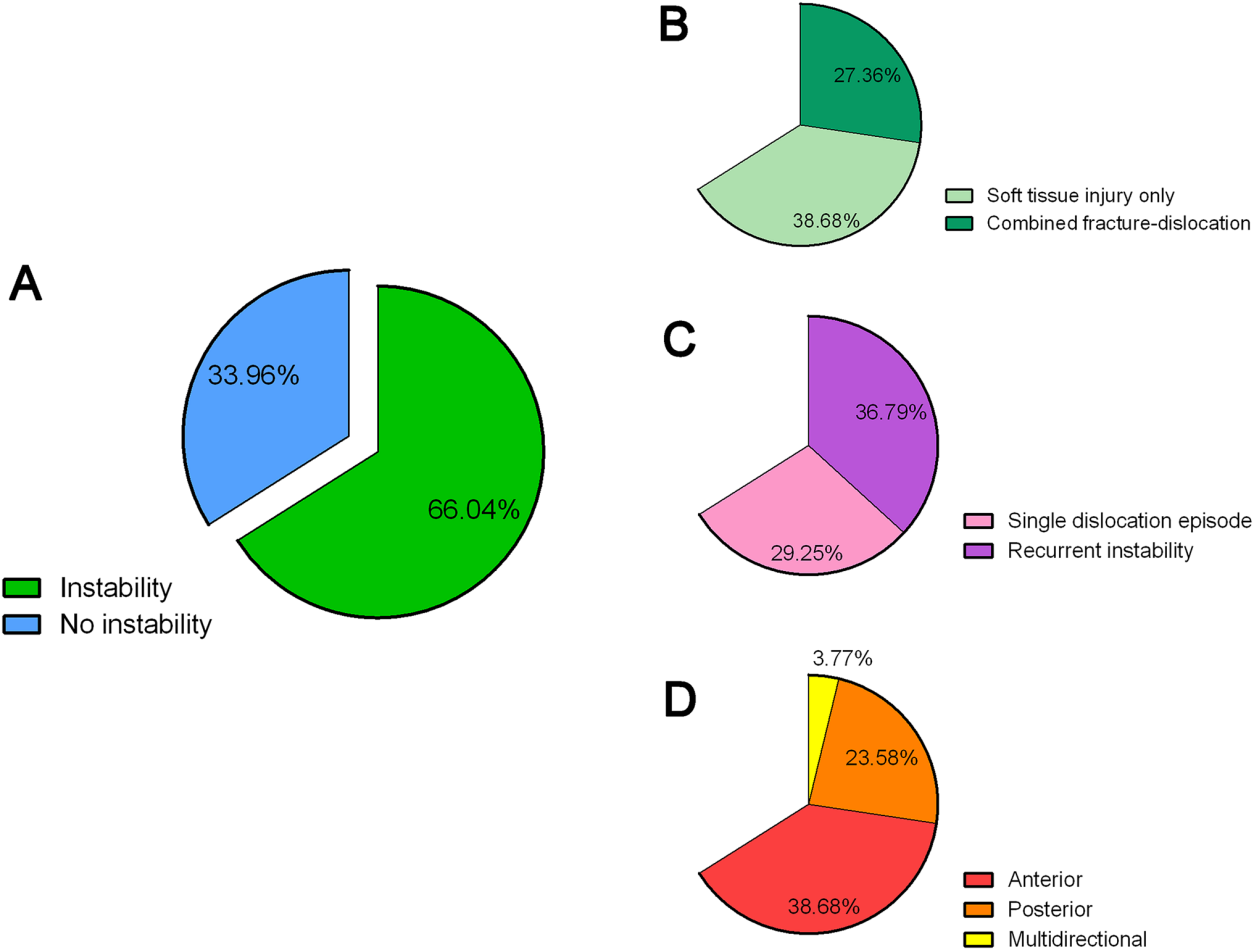
Pie charts illustrating different aspects related to shoulder instability. **A** Percentage of patients affected by shoulder instability. **B** Distribution of soft tissue and bony lesions. **C** Distribution of recurrent instability. **D** Direction of instability

The detailed description of the incidence of specific injuries (fractures, soft tissue injuries, combined and bilateral events) as well as the subgroup analysis of associations between demographic variables and different lesions are presented in Appendix A. Figure 2 and Table 2 summarize different aspects related to shoulder instability and illustrate risk factors for the development of recurrent instability. Data regarding surgical procedures and complications were available for 57 of the 64 surgically treated patients and are presented in Appendix B. The overall postoperative complication rate was 38.7%, with recurrence of instability being the most frequently reported complication (62.5%).

**Table 2:** Univariate and multivariate analyses of risk factors for the development of recurrent instability

Development of a recurrent instability	Odds ratio (95% CI)			
	Univariate	*p* value	Multivariate	*p* value
Female sex	0.346 (0.121–0.993)	**0.048**	0.289 (0.066–1.273)	0.101 (n.s.)
Older age at time of shoulder injury	0.913 (0.867–0.961)	**0.001**	0.930 (0.861–1.006)	0.069 (n.s.)
Shoulder injury during 1st seizure	0.37 (0.118–1.154)	0.087 (n.s.)		
AED at time of shoulder injury	1.714 (0.652–4.511)	0.275 (n.s.)		
Bilateral lesions	1.345 (0.421–4.293)	0.617 (n.s.)		
Anterior instability	0.36 (0.129–1.008)	0.052 (n.s.)		
Shoulder fracture-dislocation	0.103 (0.034–0.311)	** < 0.0001**	0.128 (0.035–0.462)	**0.002**
Dynamics: fall on the shoulder	0.227 (0.046–1.125)	0.069 (n.s.)		
Dynamics: muscular activation	0.550 (0.163–1.862)	0.337 (n.s.)		

Bold values indicate* p* < 0.5

*AED* antiepileptic drug; *CI* confidence interval

## Discussion

This study analysed the epidemiology, dynamics of injury, specific lesion characteristics and treatment results of a large cohort of patients suffering seizure-related shoulder injuries, confirming the extremely high frequency of bilateral injuries and posterior shoulder dislocations in this selected group of patients as compared to the general population.

Furthermore, two new relevant findings emerged from analysis of the collected data:

1. Uncontrolled muscle activation during a seizure is a distinctive dynamic of injury in patients with seizures, accounting for more than the half of all shoulder lesions, especially in younger patients. This can lead both to anterior and posterior dislocations or fracture-dislocations and is frequently cause of bilateral lesions and of instability recurrence after surgery.
2. Shoulder injury treatment in epilepsy patients is burdened by a high complication rate, especially after patients undergo surgery for instability; a recurring instability developed in almost two-thirds of those patients who underwent operative treatment.

### Shoulder injuries in epilepsy patients

When compared to the general population, epilepsy patients are more prone to suffer accidental injuries and fractures [3, 9–11], which significantly influences their quality of life [12]. Although among orthopaedic surgeons it is well known, that seizures can lead to severe shoulder injuries, more detailed knowledge on epidemiology and lesion characteristics in this selected subgroup of patients bases entirely on classic papers describing case series extracted from the records of patients treated mainly surgically at highly specialized orthopedic departments [5, 6]. This findings are confirmed in more recent literature mainly by numerous technical or clinical reports, which cannot deliver epidemiological data or information on the relative relevance of different lesions [13–19].

On the other hand, in cross-sectional studies on large cohorts of epileptic patients, shoulder injuries are rarely reported as an independent diagnosis and details on specific lesions characteristics, such as the direction of shoulder instability, are omitted [3, 4].

This study screened a large cohort of patients referring to a tertiary center for diagnosis and treatment of epilepsy and first reports the demographic characteristics of a large cohort of 106 patients, specifically investigating epidemiological aspects and differentiating the type of shoulder injuries. Here, a vast spectrum of different shoulder injuries emerged, ranging from isolated unilateral soft tissue lesions to bilateral humeral fracture-dislocations, including acute and recurrent instability in both anterior and posterior direction. Notably, the percentage of patients suffering from “rare” injuries, such as posterior dislocations and bilateral shoulder lesions was confirmed to be higher compared to the literature data from cohorts without seizures [6, 8, 14, 20–26].

Thangarajah et al. analyzed a consecutive series of 33 epileptic patients with 49 unstable shoulders who were referred to a specialized orthopaedic unit for treatment of recurrent instability and reported a mean age at the time of the index dislocation of 20 years [5]; as opposed to this, Bühler and Gerber reported a mean age of 43.5 years for 26 consecutive patients with epilepsy with 34 unstable shoulders, which comes closer to the values we encountered in our cohort (age: 39.7 ± 17.5 years) [6]. Our study revealed a difference in the average age of the patient group that experienced isolated soft tissue injuries (age: 32.4 ± 17.5 years); this was significantly lower than that for the overall patient population with shoulder injuries and, in particular, than the patient subgroup with fractures (age: 44.9 ± 15.7). In agreement with these findings, Robinson et al. noted in their series of 26 patients sustaining a complex posterior fracture dislocation, that the eleven patients who sustained an injury during a seizure were slightly younger than the remaining study patients [7]. A male dominance was documented in previous reports as well as in our study [5–7, 27].

The direction of instability differs between published reports [5, 6]. Although posterior shoulder dislocations are frequently described as being associated with seizures [6, 7, 21, 24, 27], the direction of instability in both the aforementioned patient groups was predominantly anterior [5, 6]. Our study confirmed that the dominant direction of shoulder instability in patients affected by seizures is anterior (58.6%); however, the rate of patients experiencing posterior dislocations was found to be extremely high in this selected group of patients (35.7%). Trauma dynamics did not appear to play a significant role in determining the direction of dislocation of patients who experienced uncontrolled muscle activation during a seizure: both anterior and posterior dislocations were reported.

Overall, posterior shoulder dislocations are a very rare occurrence which pose a constant challenge to the orthopaedic surgeon, since they can often be missed at initial presentation [8, 20]. The relationship between these rare types of dislocation and seizures has been previously defined [8, 28–30]. In our study, four patients experienced a posterior dislocation after directly falling on the shoulder, which confirms that powerful muscular contraction is just one of the possible injury dynamics in patients with these rare lesions.

One recommendation derived from this data collection is that a clinical examination by means of observation, palpation, passive, and active mobilisation of any painful areas should be carried out after every seizure, followed by a radiological examination of the injured regions. This is mandatory since the pain derived from fractures or dislocations can sometimes be misinterpreted for postictal muscle soreness and rhabdomyolysis as a consequence of muscle contractions [2].

Bilateral shoulder injuries are considered extremely rare and are found in literature almost always as case reports, associated with seizures [6, 14, 21–24, 27], electrocution [25], or with high-speed motor vehicle accidents or falling on both arms [26]. A recent series investigated 17 surgically treated patients who sustained a bilateral humerus fracture caused either by falling on both arms or motor vehicle accident, without history of seizures: the reported average age was 68 years, notably higher than what we encountered in our subgroup of 13 patients who sustained a bilateral humeral fracture (age: 41.5 ± 13.0 years) [26]. When considering all our 29 patients experiencing bilateral injuries, mean age dropped to 33.6 years. These data confirm that seizures are a risk factor for sustaining this rare injury also in the younger patients.

### Risk factors and treatment options

Several studies have investigated the risk factors that are associated with seizure-related injuries with seizure frequency and their generalized onset considered to be most important. Furthermore, seizure type (atonic, tonic, myoclonic, and in particular, tonic–clonic seizures), the number of antiepileptic drugs, a less independent living situation and psychiatric comorbidities have been mentioned as further, possible risk factors for seizure-related injury [12, 31–35]. Inappropriate patient positioning, such as forcing a lateral decubitus during the tonic–clonic phase of the seizure, could also increase the risk of shoulder dislocations [4].

As opposed to previously published data, drug-naïve patients with their first tonic–clonic seizure and without a prior history of epilepsy were not the only group of patients who reported a shoulder injury during a seizure [36]. Only one-quarter of our patients suffered a soft tissue shoulder injury during their first seizure and only 11% of the patients who were diagnosed with a fracture experienced it during their first seizure.

An increased fracture incidence for epilepsy patients has been described and quantified with a relative risk between 1.91 and 2.45 [3, 37–39]. Numerous risk factors explain this association: first, seizures (in particular tonic, atonic, tonic–clonic or myoclonic seizures) can lead to unprotected falls and subsequent fractures; second, uncontrolled muscle contractions during a seizure can lead to an enormous increase in the load transmitted to bones and joints, resulting in burst fractures of the spine and in joint dislocations or fracture-dislocations [40, 41]. Furthermore, some AEDs may have sedative effects increasing the risk of uncontrolled falls [42–44], whereas other reduce bone mineral density [45, 46]. In our cohort, 48% of the patients were taking AEDs when the injury occurred, which did not affect the dynamics and types of lesions. Nevertheless, the response to therapy, as well as the patients’ compliance with AEDs also plays a role in limiting seizure-related injuries [47, 48]. This aspect appeared also in our cohort, with twelve patients with epilepsy (11.3%) experiencing seizure-related shoulder injuries after discontinuing their AEDs (medical indication or non-compliance).

Treatment choice depends on the patient’s characteristics and type of shoulder injury. Regardless of the type of surgical procedure, treating shoulder injuries in epilepsy patients is difficult due to a high complication rate, with recurrence of instability occurring in almost two-thirds of patients undergoing stabilization procedures. Therefore, extreme caution is advised when planning and conducting surgery, in addition to properly consulting and educating the patient prior to surgery. Furthermore, the findings of this study highlight the role of a specialised neurological evaluation, appropriate AED treatment, and an evaluation of early epilepsy surgery or vagus nerve stimulation in drug-resistant patients. A neurologist should always be involved in treating these patients whether they present first to outpatient orthopaedic clinics or the emergency department [5].

The limitations of this study include the heterogeneous nature of the investigated cohort, with variable patients and lesions characteristics, which makes grouping and categorisation difficult. Furthermore, diagnosis and treatment of shoulder lesions occurred at different institutions, which may have different diagnostic protocols, treatment standard and orthopedic expertise, possibly leading to some variability in the lesions’ classification, decision-making, treatment choice, and complication rate. Moreover, this study did not yet collect long-term follow-up radiological data, thus not being able to show if epilepsy and seizures affect long-term radiological outcomes and osteoarthritis progression. Finally, some relevant data could neither be retrieved through examination of clinical records nor via telephonic interview; this led to some missing data and the subsequent exclusion of some patients from subgroup analysis.

## Conclusions

Epilepsy is associated with a high risk of musculoskeletal injuries, and shoulder lesions are common after seizures. Bilateral injuries and posterior dislocations, rarely observed in the general population, occur with a high frequency after seizures. The role of uncontrolled muscle activation was confirmed as a distinctive dynamic of injury in young patients with seizures, leading both to anterior and posterior dislocations and threatening the results of surgical stabilization procedures. A high complication rate was documented after treatment: this calls for appropriate preoperative counselling and a multidisciplinary approach to minimize recurring tonic–clonic seizures.

### Electronic supplementary material

Below is the link to the electronic supplementary material.

Supplementary file1 Comparison between patients with fractures (including combined fracture-dislocation episodes) and with soft tissue lesions only (DOC 35 kb)

Supplementary file2 Comparison between patients with pure anterior instability and with shoulder instability with a posterior component (posterior and multidirectional) (DOC 44 kb)

Supplementary file3 Comparison between patients with unilateral and bilateral lesions and between patients with unilateral and non-simultaneous bilateral lesions and with simultaneous bilateral lesions (DOC 57 kb)

Supplementary file4 Univariate and multivariate analyses of risk factors for the development of perioperative complications (any complication) and for the development of recurrent instability as postoperative complication (DOC 49 kb)

## Appendix A

### Fractures

Fractures around the shoulder region occurred in 60 patients (56.6% of all patients, females/males ratio: 0.32/0.68; age: 44.9 ± 15.7 years). The proximal humerus was affected in the majority of cases (53 patients, 88.3%). Combined fracture dislocations were documented in 29 cases (48.3%). The trauma dynamics for this type of injury were equally distributed between falls with a direct impact on the shoulder and muscle activation without any external impact.

Scapular fractures, including displaced fractures of the glenoid cavity, occurred in six cases (10% of all fracture patients). In all but two cases, these fractures were associated with a shoulder dislocation and a concomitant proximal humeral fracture. The trauma dynamics for this type of injury were equally distributed between falls with direct impact on the shoulder and muscle activation without any external force. Nine clavicle fractures were reported (15% of fracture patients): 5 were isolated, 3 combined with proximal humerus fractures and 1 with a glenoid fracture; 2 clavicle fractures were associated with shoulder instability.

### Bilateral proximal humeral fractures

In 13 cases, bilateral proximal humerus fractures were reported (21.7% of all fracture patients; females/males ratio: 0.23/0.77, age: 41.5 ± 13.0 years). Nine of them (69.2%) were associated with a bilateral shoulder dislocation: the direction of the dislocation was posterior in five cases and anterior in four cases. In 69.2% of the cases (nine patients), injury was caused by muscular activation, without the presence of any external acting force.

### Soft tissue injuries

Soft tissue lesions were reported in 76 patients (71.7%). The dominant trauma dynamics in patients suffering soft tissue lesions were muscular activation without action of any external impaction force (60.0% of the cases).

Capsulolabral lesions following shoulder dislocations were the most frequent type of injury pattern, which occurred in 70 patients (females/males ratio: 0.32/0.68; age 33.3 ± 14.0 years). These were associated to a single instability episode in 31 patients (44.3%), whereas recurrent instability was recorded in 39 (55.7%). The direction of instability was anterior in 41 cases (58.6%) and posterior dislocations were documented in 25 cases (35.7%). In 4 cases (5.7%), instability occurring in both anterior and posterior directions was observed at the first clinical presentation (Fig. 2). Trauma dynamics did not appear to play a significant role in determining the direction of the dislocation.

Rotator cuff lesions were documented in 17 cases only (22.4% of all patients suffering soft tissue lesions). A rotator cuff lesion combined with a shoulder dislocation was documented in 11 patients (15.7%). Univariate analysis showed that the development of a recurrent instability was influenced by sex and age at the time of shoulder injury and the presence of combined fractures. With the exception of the protective effect of an associated fracture, the sample size was insufficient to confirm these effects in a multivariate analysis (Table 2).

### Bilateral shoulder dislocations

Bilateral dislocations occurred in 24 cases (31.6% of all dislocations; females/males ratio: 0.33/0.67; age: 30.7 ± 14.4 years); in nine patients (37.5%), the bilateral dislocation was associated with a bilateral proximal humerus fracture. The direction of instability was equally distributed between purely anterior (50%) and posterior dislocations (50%). In 17 cases (70.8%), instability recurred after the first episode. Out of the 17 patients undergoing surgical treatment, 47.1% experienced recurrent instability after surgery. In all but two patients, the reported shoulder trauma dynamics were muscular activation without the presence of any external impact.

### Subgroup analysis

#### Fractures vs soft tissue lesions (Table s1)

No statistically significant differences were encountered in the percentage of male and female patients with fractures and those experiencing soft tissue lesions.

The group of patients with fractures was significantly older than those suffering from soft tissue lesions only (44.9 ± 15.7 years and 32.4 ± 17.5 years, respectively; *p* < 0.0001).

In the patient subgroup with bone injuries, the percentage of patients suffering an injury during the first seizure was lower than for those patients experiencing isolated soft tissue lesions (11% and 35%, respectively; *p* = 0.0097). However, no significant differences were detected regarding the presence of an AED at the time of shoulder injury and in the dynamics of injury.

#### Pure anterior instability vs instability with a posterior component (Table s2)

The group of patients experiencing anterior instability was significantly younger than those patients suffering instability with a posterior component (30.2 ± 12.3 years and 37.5 ± 15.1 years, respectively; *p* = 0.0329). No significant difference in terms of percentage of bilateral lesions and associated fractures and recurrence rate was encountered.

#### Unilateral vs bilateral injuries (Supplementary material, Table s3)

The group of patients with bilateral injuries was significantly younger than the patients suffering unilateral injuries at the time of shoulder injury (33.6 ± 17.2 years and 42.1 ± 17.2 years, respectively; *p* = 0.0232). No differences in terms of the percentage of patients injured during the first seizure and AED use at the time of shoulder injury were documented. A significant association between muscular activation alone as a trauma dynamic and both non-simultaneous and simultaneous bilateral injuries was documented (*p* = 0.011 and *p* = 0.0062, respectively).

Bilateral injuries were more frequently associated with instability and recurrent instability episodes (*p* = 0.0373 and *p* = 0.0135, respectively), but not to a specific direction of the instability, although dislocation with a posterior component occurred more frequently in the subgroup experiencing bilateral injuries (50%, 37% in the unilateral group). No significant differences in terms of associated fractures and combined fracture-dislocation episodes were encountered. Although the overall complication rate of both groups was comparable, patients experiencing bilateral injuries suffered more frequently from failures due to recurrent major shoulder instability episodes (40% for non-simultaneous bilateral injuries and 46% for simultaneous bilateral injuries, compared to 20% for the comparison group).

## Appendix B

### Surgical treatments and treatment results

Open reduction and internal fixation was performed in 54.4% of the cases, whereas arthroscopy was the first treatment for 29.8% of the patients; 3 patients received primary a bone block procedure for shoulder instability (two of them bilaterally). Primary joint replacement surgery was performed in 15.8% of the patients. The overall postoperative complication rate was 38.7%, with recurrence of instability being the most frequently reported complication (62.5%). Screw cut-out (1), re-fracture after new trauma (2), postoperative bleeding (1) and prosthetic failure (5) were other reported complications.

Among those patients undergoing surgery, a significantly higher percentage of failure due to recurrent major shoulder instability episodes was observed within the subgroup of patients experiencing shoulder injuries due to muscular activation (*p* = 0.011). No significant differences were observed in the overall complication rate between both trauma dynamic groups (Table 1).

A univariate analysis for the 57 patients for whom treatment results were available demonstrated that a younger age at the time of shoulder injury, injury during the first seizure, and instability are risk factors for developing complications after surgery. On the other hand, the occurrence of a shoulder fracture (including fracture/dislocation episodes) appeared to reduce the odds for developing complications after surgery. Multivariate analysis confirmed that initial instability was associated with a higher risk of developing postoperative complications (OR: 11.524, *p* = 0.032), but no other risk factors could be confirmed (Table s4). A younger age at the time of shoulder injury and arthroscopic surgery were additional risk factors for surgical failure due to recurrent, major shoulder instability in a univariate analysis; however, these results could not be confirmed in a multivariate analysis due to the limited sample size. As expected, fracture treatment appeared to be a protective factor against surgical failure due to recurrent, major shoulder instability (OR: 0.089, *p* = 0.030) (Table s4).
