# Supplementary material for: Epidemiology and specific features of shoulder injuries in patients affected by epileptic seizures
Source: Arch Orthop Trauma Surg. 2022 Mar 28;143(4):1999–2009. doi: 10.1007/s00402-022-04420-6 (PMC10030428; doi:10.1007/s00402-022-04420-6)
Supplement: Supplementary file 1 — Supplementary file1 Comparison between patients with fractures (including combined fracture-dislocation episodes) and with soft tissue lesions only (DOC 35 kb) [file 402_2022_4420_MOESM1_ESM.doc]

**Table s1: Comparison between patients with fractures (including combined fracture-dislocation episodes) and with soft tissue lesions only**

| **Group** | **Overall** | **Fractures (including combined fracture-dislocation episodes)** | **Soft tissue lesions only** | ***p-value*** |
| --- | --- | --- | --- | --- |
| **No. of patients** | **106** | **46** | **60** |  |
| **Gender (F/M ratio)** | 0.35/0.65 | 0.39/0.61 | 0.32/0.68 | *0.5378 (n.s.)* |
| **Age at time of shoulder injury (years)** | 38.00 [25.50-50.00]  39.71 ± 17.54 | 43.50 [31.25-57.75]  44.91 ± 15.71 | 29.00 [19.00-41.50]  32.43 ± 17.55 | ***<0.0001 (****)*** |
| **Shoulder injury during 1^st^ seizure (Y/N ratio)** | 0.25/0.75 | 0.11/0.89 | 0.35/0.65 | ***0.0097 (**)*** |
| **AED at time of shoulder injury (Y/N ratio)** | 0.52/0.48 | 0.45/0.55 | 0.60/0.40 | *0.2194 (n.s.)* |
| **Side (L/R ratio)** | 0.38/0.62 | 0.28/0.72 | 0.53/0.47 | ***0.0309 (*)*** |
| **Bilateral/unilateral lesions ratio** | 0.27/0.73 | 0.22/0.78 | 0.35/0.65 | *0.1869 (n.s.)* |
| **No. of patients** | **76** | **46** | **30** |  |
| **Dynamics (fall on the shoulder/ muscular activation alone ratio)** | 0.38/0.62 | 0.50/0.50 | 0.20/0.80 | ***0.0149 (*)*** |

*Continuous variables were expressed as mean ± standard deviation (SD) or as median and interquartile range (first and third quartiles, Q1-Q3), as appropriate, while the dichotomous variables are expressed in numbers of cases and frequencies. AED: antiepileptic drug; F/M: female/male; L/R: left/right; n.s.: not significant; Y/N: yes/no.*
