# Supplementary material for: Epidemiology and specific features of shoulder injuries in patients affected by epileptic seizures
Source: Arch Orthop Trauma Surg. 2022 Mar 28;143(4):1999–2009. doi: 10.1007/s00402-022-04420-6 (PMC10030428; doi:10.1007/s00402-022-04420-6)
Supplement: Supplementary file 2 — Supplementary file2 Comparison between patients with pure anterior instability and with shoulder instability with a posterior component (posterior and multidirectional) (DOC 44 kb) [file 402_2022_4420_MOESM2_ESM.doc]

**Table s2: Comparison between patients with pure anterior instability and with shoulder instability with a posterior component (posterior and multidirectional)**

| **Group** | **Overall** | **Anterior instability** | **Instability with a posterior component** | **p-value** |
| --- | --- | --- | --- | --- |
| **No. of patients** | 70 | 41 | 29 |  |
| **Gender (F/M ratio)** | 0.34/0.66 | 0.41/0.59 | 0.24/0.76 | *0.2011 (n.s.)* |
| **Age at time of shoulder injury (years)** | 31.00 [22.75-42.25]  33.32 ± 13.95 | 28.00 [22.00-38.00]  30.24 ± 12.33 | 39.00 [24.00-46.50]  37.50 ± 15.11 | ***0.0329 (*)*** |
| **Shoulder injury during 1st seizure (Y/N ratio)** | 0.25/0.75 | 0.18/0.82 | 0.34/0.66 | *0.1570 (n.s.)* |
| **AED at time of shoulder injury (Y/N ratio)** | 0.51/0.49 | 0.61/0.39 | 0.38/0.62 | *0.0866 (n.s.)* |
| **Side (L/R ratio)** | 0.46/0.54 | 0.38/0.62 | 0.59/0.41 | *0.2252 (n.s.)* |
| **Bilateral/unilateral lesions ratio** | 0.34/0.66 | 0.29/0.71 | 0.41/0.59 | *0.3179 (n.s.)* |
| **Rotator cuff tears (Y/N ratio)** | 0.16/0.84 | 0.20/0.80 | 0.10/0.90 | *0.3420 (n.s.)* |
| **Single dislocation/recurrent shoulder instability (ratio)** | 0.56/0.44 | 0.61/0.39 | 0.48/0.52 | *0.3352 (n.s.)* |
| **Any fracture (Y/N ratio)** | 0.41/0.59 | 0.39/0.61 | 0.45/0.55 | *0.8058 (n.s.)* |
| **Proximal humerus fractures (Y/N ratio)** | 0.41/0.59 | 0.39/0.61 | 0.45/0.55 | *0.8058 (n.s.)* |
| **Scapular fractures (Y/N ratio)** | 0.06/0.94 | 0.07/0.93 | 0.03/0.97 | *0.6369 (n.s.)* |
| **Clavicle fractures (Y/N ratio)** | 0.03/0.97 | 0.02/0.98 | 0.03/0.97 | *1.0000 (n.s.)* |
| **Combined shoulder fracture-dislocation (Y/N ratio)** | 0.41/0.59 | 0.39/0.61 | 0.45/0.55 | *0.8058 (n.s.)* |
| **No. of patients** | **70** | **41** | **29** |  |
| **Dynamics (fall on the shoulder/ muscular activation alone ratio)** | 0.22/0.78 | 0.25/0.75 | 0.18/0.82 | *0.7416 (n.s.)* |
| **No. of patients** | **46** | **28** | **18** |  |
| **Complications after surgery (Y/N ratio)** | 0.48/0.52 | 0.43/0.57 | 0.56/0.44 | *0.5468 (n.s.)* |
| **Recurrent shoulder instability after surgery (Y/N ratio)** | 0.33/0.67 | 0.32/0.68 | 0.33/0.67 | *1.0000 (n.s.)* |

*Continuous variables were expressed as mean ± standard deviation (SD) or as median and interquartile range (first and third quartiles, Q1-Q3), as appropriate, while the dichotomous variables are expressed in numbers of cases and frequencies. AED: antiepileptic drug; F/M: female/male; L/R: left/right; n.s.: not significant; Y/N: yes/no.*
