# Supplementary material for: Epidemiology and specific features of shoulder injuries in patients affected by epileptic seizures
Source: Arch Orthop Trauma Surg. 2022 Mar 28;143(4):1999–2009. doi: 10.1007/s00402-022-04420-6 (PMC10030428; doi:10.1007/s00402-022-04420-6)
Supplement: Supplementary file 3 — Supplementary file3 Comparison between patients with unilateral and bilateral lesions and between patients with unilateral and non-simultaneous bilateral lesions and with simultaneous bilateral lesions (DOC 57 kb) [file 402_2022_4420_MOESM3_ESM.doc]

**Table s3: Comparison between patients with unilateral and bilateral lesions and between patients with unilateral and non-simultaneous bilateral lesions and with simultaneous bilateral lesions**

| **Group** | **Overall** | **Unilateral** | **Bilateral** | **p-value** | **Unilateral and non-simultaneous bilateral** | **Simultaneous bilateral** | **p-value** |
| --- | --- | --- | --- | --- | --- | --- | --- |
| **No. of patients** | **106** | **77** | **29** |  | **89** | **17** |  |
| **Gender (F/M ratio)** | 0.35/0.65 | 0.36/0.64 | 0.31/0.69 | *0.6546 (n.s.)* | 0.35/0.65 | 0.35/0.65 | *1.0000 (n.s.)* |
| **Age at time of shoulder injury (years)** | 38.00 [25.50-50.00]  39.71 ± 17.54 | 42.00 [28.50-55.00]  42.06 ± 17.24 | 31.00 [19.00-40.00]  33.70 ± 17.15 | ***0.0232 (*)*** | 39.00 [27.00-50.00]  40.30 ± 17.09 | 32.00 [21.00-55.00]  36.94 ± 19.84 | *0.4137 (n.s.)* |
| **Shoulder injury during 1^st^ seizure (Y/N ratio)** | 0.25/0.75 | 0.24/0.76 | 0.26/0.74 | *0.7994 (n.s.)* | 0.24/0.76 | 0.29/0.71 | *0.7584 (n.s.)* |
| **AED at time of shoulder injury (Y/N ratio)** | 0.52/0.48 | 0.54/0.46 | 0.48/0.52 | *0.6577 (n.s.)* | 0.54/0.46 | 0.41/0.59 | *0.4252 (n.s.)* |
| **Rotator cuff tears (Y/N ratio)** | 0.16/0.84 | 0.14/0.86 | 0.21/0.79 | *0.5529 (n.s.)* | 0.15/0.85 | 0.24/0.76 | *0.4683 (n.s.)* |
| **Shoulder instability (Y/N ratio)** | 0.66/0.34 | 0.60/0.40 | 0.83/0.17 | ***0.0373 (*)*** | 0.62/0.38 | 0.88/0.12 | ***0.0488 (*)*** |
| **Single dislocation/recurrent shoulder instability (ratio)** | 0.64/0.36 | 0.71/0.29 | 0.45/0.55 | ***0.0135 (*)*** | 0.67/0.33 | 0.47/0.53 | *0.1660 (n.s.)* |
| **Pure anterior dislocation/ shoulder dislocation with posterior component (ratio)** | 0.59/0.41 | 0.63/0.37 | 0.50/0.50 | *0.3179 (n.s.)* | 0.62/0.38 | 0.47/0.53 | *0.3781 (n.s.)* |
| **Any fracture (Y/N ratio)** | 0.56/0.44 | 0.60/0.40 | 0.45/0.55 | *0.1869 (n.s.)* | 0.58/0.42 | 0.47/0.53 | *0.4313 (n.s.)* |
| **Proximal humerus fractures (Y/N ratio)** | 0.50/0.50 | 0.52/0.48 | 0.45/0.55 | *0.6635 (n.s.)* | 0.51/0.49 | 0.47/0.53 | *1.0000 (n.s.)* |
| **Scapular fractures (Y/N ratio)** | 0.06/0.94 | 0.06/0.94 | 0.03/0.97 | *1.0000 (n.s.)* | 0.06/0.94 | 0.06/0.94 | *1.0000 (n.s.)* |
| **Clavicle fractures (Y/N ratio)** | 0.08/0.92 | 0.09/0.91 | 0.07/0.93 | *1.0000 (n.s.)* | 0.08/0.92 | 0.12/0.88 | *0.6344 (n.s.)* |
| **Combined shoulder fracture-dislocation (Y/N ratio)** | 0.27/0.73 | 0.26/0.74 | 0.31/0.69 | *0.6301 (n.s.)* | 0.25/0.75 | 0.41/0.59 | *02331 (n.s.)* |
| **No. of patients** | **76** | **52** | **24** |  | **61** | **15** |  |
| **Dynamics (fall on the shoulder/ muscular activation alone ratio)** | 0.38/0.62 | 0.48/0.52 | 0.17/0.83 | ***0.0110 (*)*** | 0.46/0.54 | 0.07/0.93 | ***0.0062 (**)*** |
| **No. of patients** | **62** | **41** | **21** |  | **49** | **13** |  |
| **Complications after surgery (Y/N ratio)** | 0.39/0.61 | 0.39/0.61 | 0.38/0.62 | *1.0000 (n.s.)* | 0.35/0.65 | 0.46/0.54 | *0.5296 (n.s.)* |
| **Recurrent shoulder instability after surgery (Y/N ratio)** | 0.27/0.73 | 0.20/0.80 | 0.40/0.60 | *0.1225 (n.s.)* | 0.20/0.80 | 0.46/0.54 | *0.0791 (n.s.)* |

*Continuous variables were expressed as mean ± standard deviation (SD) or as median and interquartile range (first and third quartiles, Q1-Q3), as appropriate, while the dichotomous variables are expressed in numbers of cases and frequencies. AED: antiepileptic drug; F/M: female/male; L/R: left/right; n.s.: not significant; Y/N: yes/no.*
