# Supplementary material for: Epidemiology and specific features of shoulder injuries in patients affected by epileptic seizures
Source: Arch Orthop Trauma Surg. 2022 Mar 28;143(4):1999–2009. doi: 10.1007/s00402-022-04420-6 (PMC10030428; doi:10.1007/s00402-022-04420-6)
Supplement: Supplementary file 4 — Supplementary file4 Univariate and multivariate analyses of risk factors for the development of perioperative complications (any complication) and for the development of recurrent instability as postoperative complication (DOC 49 kb) [file 402_2022_4420_MOESM4_ESM.doc]

**Table s4: Univariate and multivariate analyses of risk factors for the development of perioperative complications (any complication) and for the development of recurrent instability as postoperative complication**

|  | ***Development of perioperative complications (any complication)*** | | | | ***Development of recurrent instability (as a complication)*** | | | |
| --- | --- | --- | --- | --- | --- | --- | --- | --- |
|  | **Univariate**  Odds Ratio (95% CI) | ***p-value*** | **Multivariate**  Odds Ratio (95% CI) | ***p-value*** | **Univariate**  Odds Ratio (95% CI) | ***p-value*** | **Multivariate**  Odds Ratio (95% CI) | ***p-value*** |
| **Female sex** | 1.217 (0.414 - 3.577) | *0.721 (n.s)* |  |  | 0.8 (0.241 - 2.651) | *0.715 (n.s)* |  |  |
| **Older age at time of shoulder injury** | 0.947 (0.907 - 0.990) | ***0.016*** | 0.231 (0.931 - 1.023) | *0.313 (n.s.)* | 0.855 (0.777 - 0.941) | ***0.001*** | 0.896 (0.800 - 1.003) | *0.057 (n.s.)* |
| **Shoulder injury during 1^st^ seizure** | 0.242 (0.060 - 0.971) | ***0.045*** | 0.213 (0.050 - 1.037) | *0.061 (n.s.)* | 1*^§^* |  |  |  |
| **AED at time of shoulder injury** | 1.050 (0.368 - 2.996) | *0.927 (n.s.)* |  |  | 2.095 (0.613 - 7.160) | *0.238 (n.s.)* |  |  |
| **Bilateral lesion** | 1.722 (0.483 - 6.144) | *0.402 (n.s.)* |  |  | 2.786 (0.728 - 10.655) | *0.134 (n.s.)* |  |  |
| **Shoulder instability (any type)** | 14 (1.698 - 115.415) | ***0.014*** | 11.524 (1.240 - 107.044) | ***0.032*** | 1*^§^* |  |  |  |
| **Anterior instability** | 1.055 (0.284 - 3.923) | *0.936 (n.s.)* |  |  | 0.404 (0.074 - 2.211) | *0.296(n.s.)* |  |  |
| **Any fracture** | 0.272 (0.091 - 0.817) | ***0.020*** | 0.533 (0.143 - 1.990) | *0.349 (n.s.)* | 0.037 (0.007 - 0.196) | ***< 0.0001*** | 0.089 (0.010 - 0.793) | ***0.030*** |
| **Combined shoulder fracture-dislocation** | 1.048 (0.369 - 2.975) | *0.930 (n.s.)* |  |  | 0.154 (0.031 - 0.760) | ***0.022*** | -^#^ | ***-***^#^ |
| **Dynamics: fall on the shoulder** | 0.556 (0.090 - 3.445) | *0.528 (n.s.)* |  |  | 1*^§^* |  |  |  |
| **Dynamics: muscular activation** | 1.417 (0.295 - 6.814) | *0.664 (n.s.)* |  |  | 0.8 (0.163 - 3.916) | *0.783 (n.s.)* |  |  |
| **Surgery type:** |  |  |  |  |  |  |  |  |
| **Arthroscopic** | Ref. |  |  |  | Ref. |  |  |  |
| **Open surgery** | 0.584 (0.169 - 2.017) | *0.395 (n.s.)* |  |  | 0.212 (0.051 - 0.881) | ***0.033*** | 1.438 (0.169 - 12.24) | *0.739 (n.s.)* |
| **Joint replacement** | 1.143 (0.224 - 5.841) | *0.873 (n.s.)* |  |  | 1*^§^* |  |  |  |

*AED: antiepileptic drug; CI: confidence interval.*

*^§^: Recurrent major instability after surgery developed exclusively in patients suffering from initial instability (any type), and never in patients reporting injury during 1st seizure, after falls on the shoulder or after joint replacement: for these variables, therefore, no univariate analysis was possible, which is indicated by the value of 1.*

^#^*: The variable “combined shoulder fracture-dislocation” was collinear to the variable “any fracture”, and therefore excluded from multivariate analysis.*
